# Comparative Study on Growth Index and Nutritional Quality of Female Chinese Mitten Crab *Eriocheir sinensis* Selected at Different Growth Periods in Rice-Crab Culture Systems

**DOI:** 10.1155/2023/4805919

**Published:** 2023-03-29

**Authors:** Bao-Yuan Zhang, Wen-Hao Fang, Rui Zhu, Ning Wang, Qi Yao, Hong-Jian Liu, Ji-Wu Wan, Yu-Ke Chen, Qiu-Ju Wang, Dong-Ming Zhang

**Affiliations:** ^1^College of Animal Science and Technology, Jilin Agricultural University, Jilin Changchun 130118, China; ^2^College of Life Sciences, Jilin Agricultural University, Jilin Changchun 130118, China; ^3^Aquaculture Technology Extension Station of Jilin Province, Changchun 130012, China

## Abstract

Research was conducted on the growth performance and nutritional quality of Chinese mitten crabs (*Eriocheir sinensis*) during a 62-day growing period in a symbiotic coculture comprising rice and crab. Culture experiments were conducted in three rice fields of equal size (996 m^2^). On days 0 (July 15, D0), 15 (July 30, D15), 31 (August 15, D31), 46 (August 30, D46), and 62 (September 2, D62), tissue samples of 50 female *E. sinensis* were collected randomly from each rice field. The results showed that the serum growth hormone (GH) content and muscle ecdysone receptor (*EcR*) mRNA expression levels were higher in the D31 and D46 groups; the content of serum 20-hydroxyecdysone (20-HE) and the mRNA expression levels of retinoid X receptor (*RXR*), insulin-like growth factor 2 (*IGF2*), and chitinase (*CHI*) reached the maximum in the D31 group. Muscle crude protein content gradually increased; hepatopancreas crude protein and crude lipid content began to decrease after reaching the maximum value in the D0 and D15 groups, respectively; the contents of crude protein and crude lipid in the ovary significantly increased in the D46 and D62 groups (*P* < 0.05). The content of muscle essential amino acids (EAA) reached the maximum in the D46 group; the hepatopancreas EAA content began to decrease significantly in the D31 group (*P* < 0.05); and the EAA content of the ovary decreased significantly after reaching the maximum value in the D46 group (*P* < 0.05). The muscle contents of eicosapentaenoic acid (EPA), docosapentaenoic acid (DPA), docosahexaenoic acid (DHA), polyunsaturated fatty acids (PUFA), and n-3 polyunsaturated fatty acids (n-3PUFA) and the ratio of n-3 polyunsaturated fatty acids/n-6 polyunsaturated fatty acids (n3/n6) decreased significantly in the D31 group (*P* < 0.05); the hepatopancreas contents of EPA, PUFA, n-3PUFA, and n-6 polyunsaturated fatty acids (n-6PUFA) and the ratio of n3/n6 began to decrease after reaching the maximum value in the D31 group, ethyl behenate (21:0), tetracosanoic acid (24:0), DPA, and DHA contents were detected for the first time in the D31 group; the ovary PUFA, n-3PUFA contents, and n3/n6 ratio of the D46 and D62 groups were significantly lower than those of the D31 group (*P* < 0.05). During the experimental conditions described here, female *E. sinensis* raised in rice fields reached rapid growth from August 15 to August 30. Additionally, the nutritional quality of the female *E. sinensis* edible tissues (muscle, hepatopancreas, and ovary) began to decline after August 15, when sufficient nutrients such as protein, lipid, EAA, and PUFA should be provided to the female *E. sinensis*.

## 1. Introduction

Chinese mitten crab (*Eriocheir sinensis*; *E. sinensis*) has a high economic value because of its unique taste and high nutritional value that attracts many consumers [1]. Previous studies have shown that adult female *E. sinensis* have a higher gonadal index than males and that the ovaries are a nutrient-dense edible part rich in essential amino acids and vitamins [2]. Therefore, female *E. sinensis* have a greater market prospect. Currently, *E. sinensis* is mainly farmed in intensive and high-density ponds, resulting in a deterioration of the water environment that is prone to diseases [3], such as shivering disease (caused by a combination of bacteria and viruses) [4], emulsification disease (caused by a fungal infection) [5], and hepatopancreas necrosis syndrome (caused by environmental stress) [6]. These factors led to abnormal development of the hepatopancreas and gonads, decreased contents of crude protein, fresh amino acids, and crude lipids, decreased edibility, and poor taste of *E. sinensis* [4, 5, 7, 8]. Therefore, there is an urgent need to find a green ecological model for *E. sinensis* culture.

The classic rice-crab coculture symbiosis model has ecological and environmental advantages. Currently, the rice-crab coculture system is growing strongly in China. The rice-crab coculture system is reported to provide suitable living space for *E. sinensis* while increasing rice production and economic profitability [6, 9, 10]. Meanwhile, rice-crab coculture systems have natural advantages in pest control and weed control as well as biodiversity conservation [11, 12].

However, compared with the pond culture model, the rice-crab symbiosis model has a shorter culture cycle because the stocking of *E. sinensis* should be combined with the growth of rice. Therefore, rice-crab coculture tends to result in smaller *E. sinensis* [13]. Fattening refers to feeding immature *E. sinensis* with high-nutrient feed to promote gonadal development, and reasonable fattening can effectively improve the specification and quality of rice field crabs [14]. *E. sinensis* with well-developed gonads is popular, and the price is high in China [15]. This study draws on our previous growth experiments, which were part of a larger project to investigate the developmental patterns and nutrient deposition of female *E. sinensis* under rice field farming models to discover suitable fattening time points and reasonably adjust the nutritional structure of the fattening feed. Our previous studies have observed that the rice cultivation mode has the largest specific growth rate (SGR) and weight gain rate (WGR) from July 30 to August 15 (SRG: 2.57%; WGR: 47.12%) [16]. In addition to growth performance, the nutrient deposition of the edible part of female *E. sinensis* can also be used as an important basis for exploring fattening time and adjusting fattening feed [2]. The relevant research under the rice-crab cocultivation model is relatively limited. Therefore, our study sought to investigate the regular nutrients, amino acids, and fatty acids of edible tissue (muscles, hepatopancreas, and ovary) and analyze the mRNA expression of growth-related genes of female *E. sinensis* collected at fixed intervals during the mid-late cultivation period in Gongzhuling City (Jilin Province, China). Our research results provide a reference for providing accurate fattening time and improving the nutritional quality of female *E. sinensis*. Furthermore, this study is an important guideline for the healthy culture and increased production and income of rice field crabs in Jilin Province and even in northeast China.

## 2. Materials and Methods

### 2.1. Ethical Statement

All the experimental animals used in this study were approved by the Ethics Committee of Jilin Agricultural University. All experiments were carried out in accordance with the protocols and procedures of China's “Regulations on the Management of Laboratory Animals” and approved by the Institutional Animal Care and Use Committee of Jilin Agricultural University (approval number: 20200919007).

### 2.2. Experimental Diet

The commercial feed used in this experiment was purchased from Hefeng Feed Co., Ltd., Jilin, China. The raw feed materials included fish meal, soybean meal, flour, squid paste, fish solution, soybean lecithin, fish oil, calcium dihydrogen phosphate, choline chloride, ethoxyquinoline, calcium propionate, and a mineral vitamin premix. The actual nutritional composition of feed was determined by the drying method, Kjeldahl method, Soxhlet extraction method, and 550°C burning method, respectively, as shown in Table 1.

### 2.3. Experimental Design

**Figure 1:**
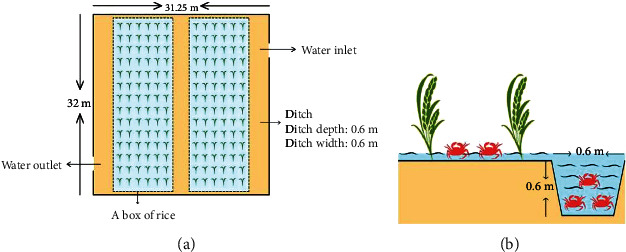
Schematic diagram showing the design of the experiment field in the rice-crab culture systems. Note: (a) floor plan of the paddy field; (b) stereoscopic view of the paddy field. Each experiment field comes equipped with a water inlet and a water outlet. Every 6 rows of rice are planted, and then 1 row is emptied to facilitate air circulation; 6 rows of rice are planted as one planting area to facilitate mechanical planting.

**Figure 2:**
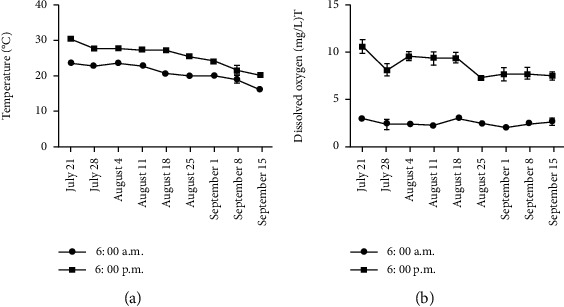
Changes of temperature and dissolved oxygen content in the rice field during *E. sinensis* culture. Note: the abscissa represents the monitored date of temperature and dissolved oxygen content. Temperature and dissolved oxygen content were monitored once a week. The data are represented as the mean ± standard deviation (*n* = 3).

The experimental site is Gongzhuling City, Jilin Province, China (longitude: 124.82°, latitude: 43.50°). Three closely connected rice fields of similar shape and size (996 m^2^), closely connected, and geographical environment were provided as three replicates by the Langu Aquaculture Farmers Professional Cooperative for *E. sinensis* culture. On both sides of the rice field, crab trenches (0.6 m^∗^0.6 m, width^∗^depth) were dug to provide living space for *E. sinensis* (Figure 1). Plastic fences are built between each rice field to prevent *E. sinensis* from escaping. Create a 90 cm high fence with plastic wrap to keep *E. sinensis* out. In addition, inlets and outlets have been installed to keep local fish and aquatic predators out of the fields. In the process of culture, the pH value in the rice field environment was in the range of 7.2-8.5, the ammonia-N was kept below 0.5 mg/L, and the changes in temperature and dissolved oxygen in the rice field were observed during the whole culture process. As illustrated in Figure 2, from groups D0 to D62, the temperature decreased. The dissolved oxygen concentration at 6:00 am was lower than at 6:00 pm In the whole breeding process, there are more sunny days, occasionally cloudy days and rain, no extreme weather. From July to September in Jilin Province, China, the weather is dry and the temperature is 16 to 32°C, which is suitable for the growth of *E. sinensis*. Juvenile *E. sinensis* were provided by Panjin Photosynthetic Crab Industry Co., Ltd. We randomly selected female *E. sinensis* that were healthy, vital, and of equal size (6.25 ± 0.77 g). According to the previous research in our laboratory [7, 8], combined with the nutritional quality and economic benefit of female *E. sinensis*, it was transferred to a rice field at a density of 0.6 crabs/m^2^. The experiment started on July 15 after the temporary breeding. During the experiment, two times a day (7:00 pm and 6:00 am), *E. sinensis* was fed 5% of its body weight. Feeding amounts should be adjusted according to the weight changes of the *E. sinensis* and the remaining feed availability. In the process of the culture experiment, the *E. sinensis* was in good health, and no abnormal behavior was observed. After the end of breeding, the ground cage was used to catch *E. sinensis*, and the average catch rate was 62.67%.

### 2.4. Sample Collection

According to the fattening time and the market time of *E. sinensis* in Jilin Province, China, on day 0 (D0: July 15), day 15 (D15: July 30), day 31 (D31: August 15), day 46 (D46: August 30), and day 62 (D62: September 15), 150 female *E. sinensis* were captured from three rice fields with similar environments (three rice fields as three repeat groups, and 50 female *E. sinensis* were captured from each rice field). Dry the surface moisture and randomly collect 5 female *E. sinensis* each repeat group to extract hemolymph from the joints of the appendages with a 1 mL sterile syringe, and immediately mix 1 : 1 with precooling anticoagulant (100 mmol L − 1 glucose, 26 mmol L − 1 citrate, 30 mmol L − 1 citric acid, 450 mmol L −1 NaCl, 10 mmol L − 1 EDTA, pH = 7.2), centrifuge for 20 minutes (9000 r/min, 4°C), then the supernatant was stored in -80 for biochemical analysis. The muscles of 5 female *E. sinensis* were collected from each replicate group, frozen in liquid nitrogen, and transferred to -80°C for growth gene expression analysis. The muscles, hepatopancreas, and ovary of 20 female *E. sinensis* were collected from each replicate group and stored at -20°C for nutritional quality analysis.

### 2.5. Analysis of Ecdysterone and Growth Hormones in Hemolymph

The contents of growth hormone (GH) and 20-hydroxyecdysone (20-HE) in the hemolymph of female *E. sinensis* were determined by an ELISA kit (Lai Er Bio-Tech, Hefei, China) of crab origin. Specifically, to the micropores coated with antibodies, samples, standards, and HRP-labeled antibodies were added successively and then incubated and washed thoroughly. Based on the principle that tetramethylbenzidine (TMB) is converted into blue under the catalysis of peroxidase and into a final yellow under the action of acid, the substrate TMB is used for color development. The depth of color is positively correlated with the content of indicators to be measured in the sample. A microplate reader was used to measure the absorbance (OD) at a wavelength of 450 nm and calculate the sample concentration.

### 2.6. Analysis of Growth Gene Expression

The expression level of mRNA of growth genes in the muscle of female *E. sinensis* followed the previous research methods of our laboratory [17] and was slightly adjusted. In short, total RNA was extracted from muscle and tested for quantity and quality, reverse transcribed into cDNA, and stored in a -80 refrigerator for RT-PCR. The detailed reflection system and cycle conditions of the RT-PCR amplification reaction are described by Zhu et al. [18]. Each sample was evaluated 3 times, and the specificity of the amplified product was determined after analyzing the dissolution curve. Specific primers are shown in Table 2, *β-actin* was used as an internal control, and the mRNA expression level of genes was analyzed by the 2^-*ΔΔ*CT^ method [19].

### 2.7. Proximate Composition Analysis

For the determination of moisture content (direct drying method), crude protein (Kjeldahl method), crude lipid (Soxhlet extraction method), and crude ash content (550°C drying method) of muscles, hepatopancreas, and ovary, specific operations refer to Chinese National Standards [20–23].

### 2.8. Amino Acid Analysis

Refer to Chinese National Standards to determine amino acids in muscle, hepatopancreas, and ovary of female *E. sinensis* [24]. Specifically, 0.1 g of the freeze-dried sample from the hydrolysis tube was transferred, and 5 mL of HCl solution (6 mol/L) was added under vacuum and then filled with nitrogen; repeat the cycle three times. Hydrolysis was performed at 110°C for 22 h. The hydrolysate was diluted to 50 mL, and 1 mL of the hydrolysate was evaporated to dryness at 50°C to remove HCl. Remains dissolved in 2 mL of water and evaporated again were then dissolved in 2 mL of sodium citrate buffer (pH = 2.2) and analyzed using an automatic amino acid analyzer (Biochrom 30, UK); calculation of amino acid concentration in the sample determination solution was calculated by an external standard method.

### 2.9. Fatty Acid Analysis

The muscle, hepatopancreas, and ovary of female *E. sinensis* were pretreated. Specifically, 2 g of tissue samples was weighed in the centrifuge tube, chloroform 20 mL and methanol 10 mL were added, sonicated for 10 minutes, and shaken for 2 hours. Then, 6 mL of NaCl aqueous solution (0.9%) was added and shaken for 30 seconds. The samples were incubated at 4°C for 22 hours and centrifuged at 3500 r/min for 10 minutes. The lower layer of chloroform was picked up after delamination. Filter paper and filtrate were placed in a dry flask and dried in a vacuum drying oven. Refer to the Chinese National Standards for saponification and derivation of lipids [25]. Analysis was performed using a gas chromatograph (Agilent 6890, USA), and the fatty acid composition of the sample was determined by the peak area normalization method [26].

### 2.10. Statistical Analyses

The experimental results are expressed as the means ± standard deviations. The data were statistically analyzed using SPSS version 20.0. The one-way ANOVA test showed that when the difference was significant (*P* < 0.05), Tukey's multiple comparison test was performed. Prism 7.00 was used for drawing.

## 3. Results

### 3.1. Growth Hormones and 20-Hydroxyecdysone in Hemolymph

**Figure 3:**
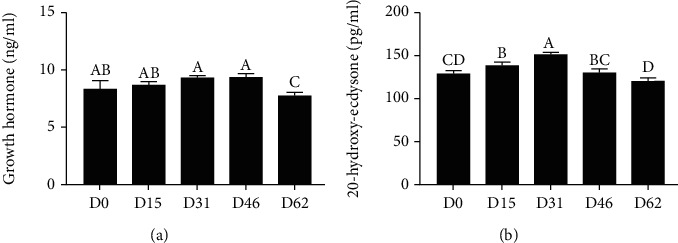
The content of growth hormone (a) and 20-hydroxyecdysone (b) in the serum of female *E. sinensis* in rice-crab culture systems at different developmental periods. ^A,B,C,D^Mean values within a row unlike superscript letters were significantly different (*P* < 0.05). Note: D0: sample data of July 15; D15: sample data of July 30; D31: sample data of August 15; D46: sample data of August 30; D62: sample data of September 15. The data are represented as the mean ± standard deviation (*n* = 3). Different letters above the columns represent significant differences observed between various treatments (*P* < 0.05).

As illustrated in Figure 3, during the growth of female *E. sinensis*, GH and 20-HE increased first and then decreased, and peaked in the D46 and D31 groups in the hemolymph, respectively. The contents of GH and 20-E in the D46 and D31 groups were significantly higher than those of the D62 group (*P* < 0.05).

### 3.2. Growth Gene Expression Analyses

**Figure 4:**
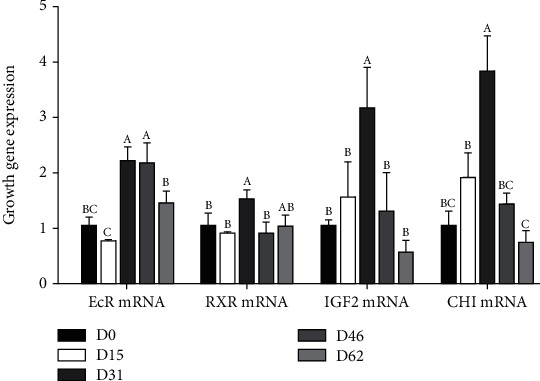
The growth gene expression levels in the muscle of female *E. sinensis* in rice-crab culture systems at different developmental periods. ^A,B,C^Mean values within a row unlike superscript letters were significantly different (*P* < 0.05). Note: D0: sample data of July 15; D15: sample data of July 30; D31: sample data of August 15; D46: sample data of August 30; D62: sample data of September 15. The data are represented as the mean ± standard deviation (*n* = 3). Different letters above the columns represent significant differences observed between various treatments (*P* < 0.05).

As illustrated in Figure 4, the muscle ecdysone receptor (*ECR*) mRNA expression levels of the D31 and D46 groups were significantly higher than those of the other groups (*P* < 0.05). The mRNA expression levels of the muscle retinoid X receptor (*RXR*) reached the highest levels in the D31 group, which value that was significantly higher than those in the D0, D15, and D46 groups (*P* < 0.05). The insulin-like growth factor 2 (*IGF2*) and chitinase (*CHI*) in muscle first increased and then decreased, and the peak appeared in the D31 group, which was significantly higher than that of the other groups (*P* < 0.05).

### 3.3. Proximate Composition Analysis

As illustrated in Table 3, in muscles, crude protein content exhibited an overall increasing trend over the course of the 62-day feeding period, while there was no significant differences were detected in moisture content (*P* > 0.05). No significant differences were detected in crude ash content in the hepatopancreas during the 62-day feeding period, while hepatopancreatic crude protein levels decreased significantly, particularly between the D31 and D62 groups (*P* < 0.05); hepatopancreatic crude lipid levels increased significantly between the D0 and D31 groups (*P* < 0.05), and then decreased slowly from the D31 to D62 groups (*P* > 0.05). In the ovaries, the levels of crude protein and crude lipid showed an upward trend, especially in the D31-D46 groups, while the difference between the D46-D62 groups was not significant during the ovary development of female *E. sinensis*.

### 3.4. Amino Acid Analysis

As illustrated in Table 4, 16 amino acids were detected in the muscle of female *E. sinensis* in the rice field. The EAA content in the ovaries first increased significantly with the feeding period to reach a peak in the D46 group prior to decreasing slightly at day 62. No significant differences were detected in lysine content, the main component of muscle EAA, during the 62-day feeding period (*P* > 0.05). Additionally, most EAA, leucine, isoleucine, threonine, valine, and TAA were significantly higher than those in the D0 group at the D31-D46 group (*P* < 0.05). The NEAA content showed an overall upward trend with the content in the D31-D61 group being significantly higher than that in the D0 group (*P* < 0.05).

As illustrated in Table 5, 16 amino acids were detected in the hepatopancreatics of female *E. sinensis* at different growth stages under rice-crab culture systems. Hepatopancreatic EAA levels increased slowly from the D0 group to the D15 group (*P* > 0.05), and then decreased significantly between the D15 group and the D62 group (*P* < 0.05). Isoleucine, leucine, and lysine, which account for a higher proportion of EAA in the hepatopancreas, have the same change trend as EAA. Some hepatopancreatic EAAs, alanine, threonine, and valine, showed an overall downward trend which was significantly lower in the D31-D62 group than in the D15 group. TAA and NEAA levels showed a trend of increasing and then decreasing, peaking in the D15 group during the 62-day breeding process.

As illustrated in Table 6, 16 amino acids were detected in the ovary of female *E. sinensis* in the rice field. The levels of EAA, NEAA, and TAA increased significantly in the D31-D46 group and decreased significantly after reaching the maximum in the D46 group (*P* < 0.05). Except for that, the changes in lysine and valine content being similar to those of EAA content, the remaining EAA decreased significantly at D31 compared with D46-D62 (*P* < 0.05). Arginine and glutamate, the main amino acid components of NEAA, began to decrease significantly in the D62 group (*P* < 0.05).

### 3.5. Fatty Acid Composition

As illustrated in Table 7, saturated fatty acids (SFAs) in female *E. sinensis* muscle showed an adult increasing trend from D0 to D46 and decreased slowly after reaching a peak in the D46 group. The monounsaturated fatty acid (MUFA) content began to increase significantly at the D31 group, which was mainly affected by the oleic acid (18:1n9c) level (*P* < 0.05). In relation to polyunsaturated fatty acids (PUFA), the linoleic acid (18:2n6c) level began to decrease significantly after reaching the peak in the D31 group (*P* < 0.05). Eicosapentaenoic acid (EPA), docosapentaenoic acid (DPA), docosahexaenoic acid (DHA), and n-3 polyunsaturated fatty acid (n-3 PUFA) levels were significantly reduced starting at the D31 group (*P* < 0.05). Likewise, the ratios of n3/n6 and DHA/EPA were significantly decreased in the D31 and D46 groups, respectively (*P* < 0.05).

As illustrated in Table 8, the SFA level showed a significant decreasing trend as a whole during the breeding process (*P* < 0.05), which was mainly affected by 16:0 (palmitic acid). The 21:0 (hexadecanoic acid) and 24:0 (tetracosanoic acid) contents of the hepatopancreas were detected for the first time in the D31 group. There was no significant difference in hepatopancreatic MUFA levels in the D0-D15 group (*P* > 0.05) until it began to decrease significantly in the D31 and D46 groups (*P* < 0.05), and then increased significantly in the D64 group (*P* < 0.05). Additionally, the 18:1n9c level was the main factor causing the change in *Σ*MUFA levels. The *Σ*PUFA level increased significantly from the D0 to the D31 group (*P* < 0.05), and then decreased significantly from the D31 to the D62 group (*P* < 0.05). DPA and DHA contents were detected for the first time in the D31 group during the 62-day culture experiment which were significantly increased from the D31 to the D46 group and then significantly decreased in the D62 group (*P* < 0.05). The n-3 PUFA, n-6 PUFA level, and n3/n6 ratio have the same horizontal change trend as the PUFA level.

As illustrated in Table 9, the level of SFA in the ovary first increased and then decreased, reaching a peak in the D46 group. 16:0, the main component of SFA, showed the same trend. The level of ovary MUFAs began to decrease significantly after reaching a maximum in the D31 group (*P* < 0.05). The contents of PUFA, n-3PUFA, n-6PUFA, the ratio of n3/n6, and DHA/EPA in the ovary reached their maximum in the D31 group. The contents of EPA in PUFA reached maximum values in the D46 group.

## 4. Discussion

Previous studies have found that GH on the endocrine growth axis plays a major role in animal growth. GH binds to growth hormone-binding protein (GHBP) and growth hormone receptor (GHR) in turn to form a GH-GHBP-GHR complex, which promotes the release of insulin-like growth factor (IGF) into the blood circulation. Additionally, IGF binds to the insulin-like growth factor binding protein (IGFBP) to form the IGF-IGFBP complex which is then transported to tissue cells of the whole body to promote the growth and differentiation of tissue cells and the growth and development of the body [27]. Our study found that the GH content of female *E. sinensis* was higher in the D31-D46 (August 15-August 30). Additionally, the fast growth rate of female *E. sinensis* at this stage was found in our previous study [16]. Similarly, Qi et al. [28] found that GH content increase can promote the weight gain of *E. sinensis*. Increased content IGF promotes the growth and differentiation of tissue cells, which increases the weight gain rate of animals [27]. In this study, the expression level of *IGF2* mRNA in female *E. sinensis* reached its peak on D31 (August 15), and the weight gain rate in our previous study also reached its maximum in the same period. Qi et al. [28] reached the same conclusion in a study of *E. sinensis*. Through the results of the GH content and *IGF2* mRNA expression in female *E. sinensis*, we speculated that the growth rate of female *E. sinensis* was faster from August 15th to August 30th under the rice field culture mode.

Crustacean molting is the basis of growth and development [29]. Ecdysteroid hormone (EH) and molting inhibitory hormone (MIH) together regulate ecdysis [30]. EH content increases with energy accumulation until ecdysone receptor (*EcR*) synthesis is induced and *EcR*-retinoid X receptor (*RXR*) heterodimers are formed [31]. 20-Hydroxyecdysone (20-E)—the active form of EH—plays a central role in regulating various physiological and behavioral changes [29, 32]. The combination of 20-E and the *EcR*-*RXR* heterodimer initiates the molt-signaling pathway to complete the molt of the body [33]. *E. sinensis* molt involves the degradation of old cuticles and the formation of new cuticles. Chitinase (*CHI*) can hydrolyze chitin in old bones. Therefore, *CHI* plays an important role in the molting process of *E. sinensis* [34]. In this experiment, female *E. sinensis* 20-E content and mRNA expression levels of *RXR* and *CHI* reached their maximum values on August 15, and the mRNA expression level of *RXR* was higher from August 15 to August 30, which was similar to the change trend of weight gain, weight gain rate, and specific growth rate of female *E. sinensis* in our previous research results, confirming that the 20-E content was closely related to the growth and development. Similarly, Chen et al. [35] found that the higher the expression levels of *EcR* and *RXR* were, the greater the weight gain rate.

In addition, previous studies have reported that 20-E plays an important role in the development and reproduction of arthropods [36, 37]. Similarly, the content of 20-E reached its maximum in the early stage of ovarian development in our study. However, this experiment found that the 20-E content gradually decreased with the maturation of the ovary, and similar results were obtained in the study of *Scylla paramamosain* [38]. Some studies suggest that 20-E may be converted into other forms of ecdysone during late ovarian maturation. Gong [38] speculated that the large accumulation of 20-E in the early stage of ovary development completely promote subsequent ovarian development. Another reason may be that the ovary can synthesize EH and convert it to 20-E at the later stage of ovarian maturation, while the Y organ synthesizes less EH that was transported to the ovary by hemolymph [38]. However, the specific mechanism needs to be further studied. Previous studies have found the 20-E and *EcR*-*RXR* heterodimer complex can promote ovarian development by regulating vitellogenin [31]. In the study of *Oziotelphusa senex senex*, the upregulation of *EcR* and *RXR* mRNA expression levels induces the synthesis of vitellogenin, which enters the blood and is absorbed by the ovary, thereby promoting the synthesis of yolk [39]. Similar results have been found in *Metapenaeus ensis* studies. In this experiment, the ovaries of female *E. sinensis* began to appear on August 15, when the expression levels of *EcR* and *RXR* mRNA reached the maximum, which was basically consistent with the ovarian development of female *E. sinensis* [16].

The approximate composition of edible tissue (muscle, hepatopancreas, and ovary) of female *E. sinensis* can reflect the deposition level of the body's nutrients, which are the reference for evaluating the nutritional value and time to market of *E. sinensis*. Previous studies have found that the larger the size of *Procambarus clarkii* and *Eriocheir sinensis*, the higher the muscle protein content [16, 40]. Similarly, our study demonstrated that the crude protein content of female *E. sinensis* muscle increases with growth, which revealed that the muscle protein of female *E. sinensis* also accumulates during the growth process. The hepatopancreas is the organ of lipid storage and metabolism in crustaceans [41]. The results showed that compared with muscles and ovaries, the hepatopancreas of female *E. sinensis* had a higher crude lipid content. It is worth noting that the crude protein and crude lipid contents of the female *E. sinensis* hepatopancreas began to decline on August 15. Previous studies have found that the hepatopancreas is the nutrient storage organ of female *E. sinensis*, and when the ovaries develop rapidly, nutrients are transferred from the hepatopancreas to the ovaries [2]. Previous studies in our laboratory found that the hepatopancreatic index (HSI) and gonadal index (GSI) of female *E. sinensis* were negatively correlated under the rice-field culture mode, which confirmed this phenomenon. No significant changes in ovarian crude protein and crude lipid content from August 30 to September 15 were found in our study, while our previous research found that female *E. sinensis* GSI increased after the appearance of ovaries. However, Long et al. [2] studies found that improving feed nutrition and timely fattening could increase the contents of crude protein and crude lipid in the ovaries of *E. sinensis* during fattening. In the culture of this experiment, the nutrition supplied by feed to female *E. sinensis* remained unchanged, which may lead to an insufficient nutrition supply for ovarian growth of female *E. sinensis* during the late stage of breeding. We speculate that this may be one of the potential factors leading to the decrease in hepatopancreatic nutritional quality and ovarian development of female *E. sinensis* in rice fields; additionally, during the period of rapid ovarian development of female *E. sinensis* (August 15-September 15), high-nutrient feed should be replaced. However, under the cultivation of *E. sinensis* in northern rice fields, most *E. sinensis* were supplied with the same nutrient composition feed throughout the culture process or did not even give feed and were completely dependent on natural bait in the rice field environment. Our research results further confirm that this breeding and feeding method was unreasonable.

Protein is the most important energy source of crustaceans and is composed of a variety of amino acids; in addition, amino acid composition is also an index that reflects the nutritional value of *E. sinensis* [42, 43]. Under the rice field culture mode, the contents of fresh amino acids (glutamic acid and aspartic acid) were higher in the edible tissues of female *E. sinensis*, and the ratio of EAA/TAA was approximately 0.4 (the best ratio of EAA/TAA), which further confirms that rice field crabs were delicious and nutritious [44]. Adequate total amino acids are not only the basis for maintaining body growth and health but also the embodiment of nutritional value [45]. In our study, the content of EAA in female *E. sinensis* muscle increased at first and then decreased, and was higher from August 15 to August 30, which was mainly dominated by isoleucine, leucine, threonine, and valine. Previous studies have found that isoleucine, leucine, and valine as branched chain amino acids can promote the release of insulin and GH by the body to accelerate body growth [46]. Our study found that the contents of isoleucine, leucine, and valine in the muscle of female *E. sinensis* were higher from August 15 to August 30, which was consistent with the change trend of serum GH content. EAA plays an important role in the ovarian development of female *E. sinensis*. However, in our study, the contents of TAA and EAA in the hepatopancreas of female *E. sinensis* decreased from August 15 to September 15. Similarly, the contents of isoleucine, leucine, lysine, phenylalanine, threonine, and valine in EAA showed similar trends. Low nutrition of feed may result in the deficiency of amino acids in female *E. sinensis* in rice fields at the later stage of culture, which needs to be supplemented with a proper amount of EAA. In addition, the flow of nutrients from the hepatopancreas to the ovaries results in a decrease in the content of EAA in the hepatopancreas [47]. Our study found that with the appearance of female *E. sinensis* ovaries, the EAA content in the ovary increased gradually from August 15 to August 30, while the EAA content in the hepatopancreas decreased gradually. During the rapid development of female *E. sinensis* ovaries, the nutrients stored in the hepatopancreas were transferred to the ovaries for ovarian development. In addition, Long et al. [2] found that the content of EAA in ovaries increased gradually with the development of the ovaries with adequate nutrition. However, in this experiment, the contents of TAA, EAA, lysine, and valine in the ovaries of female *E. sinensis* decreased from August 30 to September 15, which may be due to whether the female *E. sinensis* were fattened or not. Under the classical pond culture mode, fattening feed with a higher nutritional value needs to be replaced in the later stage of culture to meet the hepatopancreas and gonadal growth of *E. sinensis*. However, during the later stage of breeding, the amino acid demand of female *E. sinensis* in the rice field was not met, which consumed more hepatopancreatic nutrition to supply ovarian development in this experiment. Therefore, under the mode of rice field culture, the content of EAA in the feed of female *E. sinensis* should be properly increased from August 15 to September 15.

Fatty acid composition, as an important index to evaluate the nutritional quality of *E. sinensis*, plays an important role in the growth and physiological processes of *E. sinensis* [2]. In our study, the n3/n6 ratios of edible tissues in different periods were all above the 0.1-0.2 recommended by FAO/WHO, indicating that female *E. sinensis* have high nutritional value [44]. The main energy supply fatty acid of *E. sinensis* was SFA. PUFAs contain a variety of essential fatty acids, which play an important role in regulating the growth and development, molting, and immune function of *E. sinensis* [48, 49]. In our study, 7 kinds of SFAs, 5 kinds of MUFAs, and 11 kinds of PUFAs were detected in female *E. sinensis* muscles, and the contents of SFAs and MUFAs in female *E. sinensis* muscles from August 15 to September 15 were higher than those from July 15 to July 30. Previous studies have shown that the content of MUFA in the muscle of *Scylla serrata* increases when the dietary essential fatty acids are insufficient [50]. Under rice field culture, we observed that the contents of PUFA, n-3PUFA, EPA, DPA, and DHA in the muscle of female *E. sinensis* decreased from August 15 to September 15, which suggests that there may be a deficiency of essential fatty acids during this period. Similarly, Tian et al. [51] showed that reduced dietary lipid content significantly increased MUFA content and decreased PUFA content in the muscle of *Oreochromis niloticus*, which supported our results. Similar to muscle, the contents of PUFA, n-3PUFA, n-6PUFA, and EPA in the hepatopancreas of female *E. sinensis* in the rice field reached a maximum on August 15 and then began to decrease. The decrease in the content of *β*-oxidized main energy supply fatty acid SFA in the hepatopancreas of female *E. sinensis* from August 15 to September 15 indicated that the body's energy consumption increased and lipid supply was insufficient [48, 49]. In addition, the reduction in PUFA content in the hepatopancreas may be related to lipid transfer [47]. Increases in gonadal proteins and fatty acids during gonadal development in most crustaceans were directly related to decreases in proteins and fatty acids in the hepatopancreas [2, 52]. In the present study, the ovaries of female *E. sinensis* in rice fields were first observed on August 15 and developed rapidly, which required a large amount of PUFA [16]. However, crustaceans have a limited ability to synthesize PUFAs [53]. In our study, the female *E. sinensis* were not fed a fattening diet during ovary development, and the decrease in the content of crude lipids and PUFA in the hepatopancreas revealed that fatty acid transfer in the hepatopancreas may be the basis for the rapid development of ovaries. In addition, Yuan et al. [54] pointed out that the addition of lecithin and fish oil to feed could significantly promote the gonadal development of *E. sinensis*. In particular, our study found that the contents of DPA, PUFA, and n-3PUFA and the ratio of n3/n6 in the ovaries of female *E. sinensis* decreased on August 30, while EPA decreased on September 15, which may be caused by lipid deficiency. The specific reasons need to be further studied.

## 5. Conclusion

According to our research, the level of growth-related hormones and gene expression of female *E. sinensis* cultured under rice-crab culture systems reached the peaked between August 15 and August 30. The nutritional quality of edible tissue increased from July 15 to July 30, and then the levels of protein and amino acids in muscle tended to smooth while the content of PUFA decreased from August 15 to September 15. The nutritional quality of the hepatopancreas and ovary began to decrease after August 15 and August 30, respectively. There are differences in the nutrient deposition capacity of each edible tissue at different stages. We speculate that this is closely related to the nutritional requirements of female crabs in rice fields. The nutrient content requirements of female *E. sinensis* in rice fields should be the focus of future research at different stages.

## Figures and Tables

**Table 1:** Nutrition level of experimental diet (%).

Diet nutrition level	
Moisture	11.67%
Crude protein	30.16%
Crude lipids	6.03%
Crude ash	16.52%

Note: the commercial feed used in this experiment was purchased from Hefeng Feed Co., Ltd., Jilin, China. The feed raw materials include fish meal, soybean meal, flour, squid paste, fish solution, soybean lecithin, fish oil, calcium dihydrogen phosphate, choline chloride, ethoxyquinoline, calcium propionate, and mineral vitamin premix.

**Table 2:** Primer sequences used for real-time PCR.

Genes	Position	5′-3′ primer sequence	Access no.
*EcR*	Forward	GGGCATCGGGCTACCACTACAAC	KC823045.1
*EcR*	Reverse	GGCACTGAGACTCGGGCACAACA	
*RXR*	Forward	ACTGCTGCAATGACGTGGAA	KF179131
*RXR*	Reverse	GCTCGTCAGGGTAGGTGGTG	
*IGF2*	Forward	AAGTCCTGGAAAGCGTTGG	XM015444761.1
*IGF2*	Reverse	GTCACATGCACCACCTGTGT	
*CHI*	Forward	GAGCCCTACGTCTACAGCATCAC	KJ513466.1
*CHI*	Reverse	GGTCTCAACACTCCAAACCATCA	
*β-Actin*	Forward	TGGGTATGGAATCCGTTGGC	KM244725.1
*β-Actin*	Reverse	AGACAGAACGTTGTTGGCGA	

**Table 3:** Approximate composition of female *E. sinensis* in rice-crab coculture system at different developmental periods (% fresh weight).

Tissue	Group				
	D0	D15	D31	D46	D62
Muscle					
Moisture	80.73 ± 1.16^a^	79.95 ± 0.48^a^	79.57 ± 2.40^a^	79.43 ± 0.40^a^	78.67 ± 1.42^a^
Crude protein	15.25 ± 0.13^b^	16.03 ± 0.15^ab^	16.45 ± 0.53^ab^	16.95 ± 1.05^ab^	17.29 ± 1.01^a^
Crude lipids	1.45 ± 0.13^a^	1.48 ± 0.10^a^	1.48 ± 0.06^a^	1.41 ± 0.11^a^	1.46 ± 0.06^a^
Crude ash	1.47 ± 0.05^a^	1.56 ± 0.11^a^	1.55 ± 0.15^a^	1.58 ± 0.14^a^	1.61 ± 0.15^a^
Hepatopancreas					
Moisture	53.36 ± 0.21^bc^	50.40 ± 1.40^d^	51.09 ± 0.28^cd^	55.52 ± 1.14^ab^	56.06 ± 0.54^a^
Crude protein	10.15 ± 0.22^a^	10.06 ± 0.28^a^	9.09 ± 0.35^b^	6.14 ± 0.20^c^	6.12 ± 0.29^c^
Crude lipids	33.81 ± 0.98^b^	37.29 ± 1.22^a^	37.10 ± 0.53^a^	35.37 ± 1.08^ab^	34.57 ± 1.41^ab^
Crude ash	0.95 ± 0.03^a^	1.03 ± 0.04^a^	0.93 ± 0.02^a^	0.97 ± 0.06^a^	0.94 ± 0.05^a^
Ovary					
Moisture	—	—	77.52 ± 1.08^a^	70.19 ± 2.28^b^	70.15 ± 0.41^b^
Crude protein	—	—	13.91 ± 0.36^b^	15.99 ± 0.26^a^	16.04 ± 0.56^a^
Crude lipids	—	—	4.19 ± 0.04^b^	9.55 ± 0.18^a^	9.87 ± 0.18^a^
Crude ash	—	—	2.01 ± 0.10^a^	2.20 ± 0.05^a^	2.15 ± 0.17^a^

Note: the data are represented as the mean ± standard deviation (*n* = 3). Values in the same row with different letters are significantly different (*P* < 0.05). “-” represents no data for this group.

**Table 4:** Amino acid composition of female *E. sinensis* muscle in rice-crab coculture system at different developmental periods (% wet weight).

Amino acid composition	Group				
	D0	D15	D31	D46	D62
Isoleucine	0.61 ± 0.03^b^	0.64 ± 0.01^ab^	0.67 ± 0.02^a^	0.68 ± 0.01^a^	0.66 ± 0.02^ab^
Leucine	1.05 ± 0.05^b^	1.08 ± 0.04^ab^	1.12 ± 0.02^a^	1.13 ± 0.01^a^	1.13 ± 0.02^a^
Lysine	1.19 ± 0.04^a^	1.16 ± 0.02^a^	1.18 ± 0.07^a^	1.22 ± 0.01^a^	1.20 ± 0.02^a^
Methionine	0.38 ± 0.03^a^	0.32 ± 0.03^a^	0.32 ± 0.06^a^	0.36 ± 0.01^a^	0.30 ± 0.06^a^
Phenylalanine	0.63 ± 0.01^b^	0.68 ± 0.02^a^	0.69 ± 0.02^a^	0.67 ± 0.01^ab^	0.69 ± 0.03^a^
Tyrosine	0.64 ± 0.02^b^	0.67 ± 0.02^ab^	0.72 ± 0.01^a^	0.67 ± 0.02^ab^	0.67 ± 0.02^ab^
Threonine	0.62 ± 0.02^b^	0.66 ± 0.00^ab^	0.68 ± 0.02^a^	0.69 ± 0.01^a^	0.69 ± 0.02^a^
Valine	0.65 ± 0.02^b^	0.69 ± 0.00^ab^	0.71 ± 0.02^a^	0.70 ± 0.01^a^	0.70 ± 0.03^a^
EAA	5.78 ± 0.10^b^	5.90 ± 0.04^ab^	6.08 ± 0.10^a^	6.12 ± 0.02^a^	6.04 ± 0.19^ab^
Histidine	0.38 ± 0.01^b^	0.41 ± 0.01^a^	0.39 ± 0.01^ab^	0.40 ± 0.00^ab^	0.42 ± 0.01^a^
Arginine	1.47 ± 0.06^a^	1.50 ± 0.01^a^	1.56 ± 0.06^a^	1.57 ± 0.01^a^	1.53 ± 0.03^a^
Glutamic acid	2.34 ± 0.13^a^	2.37 ± 0.05^a^	2.43 ± 0.10^a^	2.39 ± 0.00^a^	2.43 ± 0.03^a^
Aspartic acid	1.38 ± 0.06^a^	1.43 ± 0.02^a^	1.44 ± 0.05^a^	1.47 ± 0.00^a^	1.47 ± 0.02^a^
Alanine	1.01 ± 0.03^c^	1.19 ± 0.02^ab^	1.15 ± 0.05^ab^	1.22 ± 0.00^a^	1.14 ± 0.02^b^
Glycine	0.85 ± 0.03^c^	0.90 ± 0.01^bc^	0.95 ± 0.02^b^	0.91 ± 0.01^b^	1.01 ± 0.03^a^
Serine	0.58 ± 0.02^b^	0.59 ± 0.01^b^	0.62 ± 0.02^ab^	0.64 ± 0.02^a^	0.62 ± 0.02^ab^
Proline	0.58 ± 0.01^d^	0.65 ± 0.01^c^	0.65 ± 0.01^c^	0.72 ± 0.04^b^	0.77 ± 0.01^a^
NEAA	8.59 ± 0.33^b^	9.03 ± 0.03^ab^	9.19 ± 0.30^a^	9.32 ± 0.06^a^	9.39 ± 0.18^a^
TAA	14.37 ± 0.41^b^	14.93 ± 0.02^ab^	15.27 ± 0.38^a^	15.43 ± 0.07^a^	15.42 ± 0.37^a^
EAA/TAA	0.40 ± 0.01^a^	0.39 ± 0.01^a^	0.40 ± 0.01^a^	0.40 ± 0.01^a^	0.39 ± 0.01^a^

Note: the data are represented as the mean ± standard deviation (*n* = 3). Values in the same row with different letters are significantly different (*P* < 0.05). EAA: essential amino acids; NEAA: nonessential amino acids; TAA: total amino acids.

**Table 5:** Amino acid composition of female *E. sinensis* hepatopancreas in rice-crab coculture system at different developmental periods (% wet weight).

Amino acid composition	Group				
	D0	D15	D31	D46	D62
Isoleucine	0.41 ± 0.02^b^	0.44 ± 0.01^a^	0.35 ± 0.01^c^	0.23 ± 0.00^e^	0.30 ± 0.02^d^
Leucine	0.72 ± 0.02^a^	0.76 ± 0.01^a^	0.60 ± 0.01^b^	0.42 ± 0.01^c^	0.46 ± 0.04^c^
Lysine	0.61 ± 0.02^a^	0.64 ± 0.02^a^	0.52 ± 0.01^b^	0.39 ± 0.01^c^	0.34 ± 0.01^d^
Methionine	0.17 ± 0.01^a^	0.18 ± 0.01^a^	0.15 ± 0.04^ab^	0.11 ± 0.01^bc^	0.07 ± 0.00^c^
Phenylalanine	0.54 ± 0.02^a^	0.47 ± 0.01^b^	0.45 ± 0.01^c^	0.29 ± 0.01^d^	0.29 ± 0.01^d^
Tyrosine	0.44 ± 0.02^a^	0.42 ± 0.01^a^	0.41 ± 0.01^a^	0.28 ± 0.03^b^	0.28 ± 0.02^b^
Threonine	0.54 ± 0.01^a^	0.54 ± 0.02^a^	0.49 ± 0.03^b^	0.34 ± 0.01^c^	0.32 ± 0.00^c^
Valine	0.55 ± 0.02^a^	0.51 ± 0.01^b^	0.46 ± 0.02^c^	0.33 ± 0.02^d^	0.32 ± 0.01^d^
EAA	4.21 ± 0.10^a^	4.22 ± 0.05^a^	3.64 ± 0.09^b^	2.52 ± 0.04^c^	2.52 ± 0.07^c^
Histidine	0.23 ± 0.01^ab^	0.25 ± 0.00^a^	0.21 ± 0.01^b^	0.13 ± 0.02^c^	0.15 ± 0.02^c^
Arginine	0.65 ± 0.04^a^	0.68 ± 0.02^a^	0.65 ± 0.05^a^	0.44 ± 0.04^b^	0.44 ± 0.03^b^
Glutamic acid	1.35 ± 0.08^a^	1.45 ± 0.05^a^	1.15 ± 0.08^b^	0.65 ± 0.03^c^	0.67 ± 0.02^c^
Aspartic acid	1.04 ± 0.04^a^	1.11 ± 0.01^a^	0.99 ± 0.09^a^	0.64 ± 0.04^b^	0.54 ± 0.01^b^
Alanine	0.56 ± 0.03^ab^	0.58 ± 0.02^a^	0.52 ± 0.02^b^	0.40 ± 0.01^c^	0.38 ± 0.01^c^
Glycine	0.52 ± 0.02^a^	0.57 ± 0.02^a^	0.56 ± 0.05^a^	0.36 ± 0.01^b^	0.32 ± 0.02^b^
Serine	0.44 ± 0.05^a^	0.34 ± 0.01^bc^	0.40 ± 0.01^ab^	0.28 ± 0.01^cd^	0.25 ± 0.02^d^
Proline	0.33 ± 0.01^c^	0.48 ± 0.02^a^	0.43 ± 0.03^b^	0.30 ± 0.01^c^	0.33 ± 0.01^c^
NEAA	5.10 ± 0.19^ab^	5.45 ± 0.03^a^	4.89 ± 0.24^b^	3.20 ± 0.09^c^	3.08 ± 0.06^c^
TAA	9.08 ± 0.28^a^	9.42 ± 0.03^a^	8.32 ± 0.33^b^	5.59 ± 0.10^c^	5.45 ± 0.09^c^
EAA/TAA	0.44 ± 0.01^a^	0.42 ± 0.01^ab^	0.41 ± 0.01^b^	0.43 ± 0.01^ab^	0.43 ± 0.01^ab^

Note: the data are represented as the mean ± standard deviation (*n* = 3). Values in the same row with different letters are significantly different (*P* < 0.05). EAA: essential amino acids; NEAA: nonessential amino acids; TAA: total amino acids.

**Table 6:** Amino acid composition of female *E. sinensis* ovary in rice-crab coculture system at different developmental periods (% wet weight).

Amino acid composition	Group		
	D31	D46	D62
Isoleucine	0.55 ± 0.01^b^	0.79 ± 0.02^a^	0.76 ± 0.01^a^
Leucine	1.02 ± 0.02^b^	1.33 ± 0.03^a^	1.30 ± 0.01^a^
Lysine	1.01 ± 0.01^c^	1.17 ± 0.02^a^	1.07 ± 0.03^b^
Methionine	0.32 ± 0.01^b^	0.51 ± 0.01^a^	0.49 ± 0.01^a^
Phenylalanine	0.71 ± 0.02^b^	0.92 ± 0.02^a^	0.90 ± 0.01^a^
Tyrosine	0.68 ± 0.01^b^	0.90 ± 0.02^a^	0.91 ± 0.02^a^
Threonine	0.73 ± 0.02^b^	0.97 ± 0.02^a^	0.97 ± 0.01^a^
Valine	0.80 ± 0.01^c^	1.08 ± 0.01^a^	1.03 ± 0.01^b^
EAA	5.81 ± 0.07^c^	7.66 ± 0.11^a^	7.43 ± 0.03^b^
Histidine	0.43 ± 0.01^a^	0.42 ± 0.01^a^	0.42 ± 0.02^a^
Arginine	1.35 ± 0.01^a^	1.30 ± 0.03^a^	1.23 ± 0.01^b^
Glutamic acid	2.16 ± 0.01^a^	2.18 ± 0.02^a^	2.10 ± 0.01^b^
Aspartic acid	1.26 ± 0.01^b^	1.50 ± 0.04^a^	1.48 ± 0.04^a^
Alanine	0.85 ± 0.01^a^	0.88 ± 0.01^a^	0.85 ± 0.03^a^
Glycine	0.96 ± 0.01^a^	0.75 ± 0.01^b^	0.73 ± 0.01^c^
Serine	0.67 ± 0.01^b^	0.99 ± 0.02^a^	0.97 ± 0.01^a^
Proline	0.83 ± 0.01^a^	0.88 ± 0.02^a^	0.86 ± 0.04^a^
NEAA	8.51 ± 0.02^b^	8.90 ± 0.06^a^	8.63 ± 0.11^b^
TAA	14.32 ± 0.08^c^	16.57 ± 0.17^a^	16.06 ± 0.13^b^
EAA/TAA	0.41 ± 0.01^b^	0.46 ± 0.00^a^	0.46 ± 0.01^a^

Note: the data are represented as the mean ± standard deviation (*n* = 3). Values in the same row with different letters are significantly different (*P* < 0.05). EAA: essential amino acids; NEAA: nonessential amino acids; TAA: total amino acids.

**Table 7:** Fatty acid composition of female *E. sinensis* muscle in rice-crab coculture system at different developmental periods (% dry weight).

Fatty acid composition	Group				
	D0	D15	D31	D46	D62
12:0	0.19 ± 0.09^ab^	0.13 ± 0.03^b^	0.14 ± 0.08^b^	0.17 ± 0.03^ab^	0.35 ± 0.10^a^
14:0	0.57 ± 0.01^b^	0.57 ± 0.16^b^	1.23 ± 0.26^a^	1.07 ± 0.32^ab^	0.63 ± 0.13^b^
15:0	0.33 ± 0.00^a^	0.36 ± 0.01^a^	0.50 ± 0.04^a^	0.54 ± 0.21^a^	0.35 ± 0.05^a^
16:0	17.55 ± 1.28^b^	18.13 ± 0.93^b^	21.84 ± 1.0^a^	23.27 ± 1.47^a^	23.29 ± 0.32^a^
17:0	1.62 ± 0.04^a^	1.60 ± 0.20^a^	1.04 ± 0.06^b^	0.83 ± 0.08^b^	1.07 ± 0.30^b^
18:0	8.36 ± 0.52^a^	7.77 ± 0.67^a^	6.63 ± 0.24^a^	6.62 ± 0.36^a^	6.67 ± 1.50^a^
20:0	0.30 ± 0.06^a^	0.25 ± 0.01^a^	0.31 ± 0.06^a^	0.30 ± 0.04^a^	0.25 ± 0.06^a^
SFA	28.91 ± 0.12^b^	28.79 ± 0.18^b^	31.70 ± 1.51^ab^	32.79 ± 2.34^a^	32.62 ± 1.51^ab^
16:1	3.01 ± 0.08^b^	3.07 ± 1.21^b^	5.07 ± 0.08^a^	5.33 ± 0.67^a^	5.56 ± 0.80^a^
17:1	0.47 ± 0.10^a^	0.34 ± 0.04^a^	0.51 ± 0.08^a^	0.51 ± 0.19^a^	0.49 ± 0.09^a^
18:1n9t	0.29 ± 0.01^a^	0.22 ± 0.03^b^	0.32 ± 0.03^a^	0.32 ± 0.05^a^	0.32 ± 0.00^a^
18:1n9c	21.86 ± 0.71^c^	22.08 ± 0.43^bc^	25.99 ± 1.74^a^	25.76 ± 0.95^a^	24.60 ± 1.21^ab^
20:1n9	0.52 ± 0.01^a^	0.48 ± 0.06^a^	0.19 ± 0.04^b^	0.15 ± 0.04^b^	0.17 ± 0.03^b^
MUFA	26.16 ± 0.71^b^	26.19 ± 0.88^b^	32.08 ± 1.76^a^	32.08 ± 1.53^a^	31.34 ± 1.69^a^
18:2n6c	13.02 ± 0.14^b^	13.74 ± 0.18^b^	16.79 ± 1.34^a^	13.72 ± 0.65^b^	13.99 ± 0.44^b^
18:3n6	0.37 ± 0.02^a^	0.39 ± 0.13^a^	0.53 ± 0.25^a^	0.56 ± 0.21^a^	0.48 ± 0.17^a^
18:3n3	4.11 ± 0.01^ab^	4.23 ± 0.39^a^	3.10 ± 0.54^bc^	3.61 ± 0.63^abc^	2.93 ± 0.10^c^
20:2	0.88 ± 0.16^a^	1.00 ± 0.27^a^	0.25 ± 0.02^b^	0.25 ± 0.05^b^	1.07 ± 0.08^a^
20:3n6	0.86 ± 0.04^a^	0.90 ± 0.10^a^	0.81 ± 0.12^ab^	1.02 ± 0.30^a^	0.43 ± 0.02^b^
20:3n3	0.55 ± 0.02^a^	0.54 ± 0.04^a^	0.53 ± 0.04^a^	0.60 ± 0.05^a^	0.42 ± 0.02^b^
20:4n6	9.13 ± 0.21^a^	9.19 ± 0.08^a^	7.41 ± 0.59^a^	7.60 ± 1.57^a^	7.67 ± 0.60^a^
20:5n3 (EPA)	10.97 ± 0.35^a^	10.29 ± 1.16^a^	4.77 ± 0.50^b^	5.58 ± 0.78^b^	6.76 ± 0.72^b^
22:2	0.38 ± 0.07^a^	0.37 ± 0.00^a^	0.23 ± 0.01^b^	0.30 ± 0.05^ab^	0.26 ± 0.07^ab^
22:5n3 (DPA)	0.74 ± 0.05^a^	0.71 ± 0.05^a^	0.43 ± 0.03^b^	0.41 ± 0.02^b^	0.37 ± 0.04^b^
22:6n3 (DHA)	3.92 ± 0.19^a^	3.66 ± 0.40^a^	1.37 ± 0.05^b^	1.49 ± 0.07^b^	1.67 ± 0.27^b^
PUFA	44.93 ± 0.08^a^	45.02 ± 1.05^a^	36.22 ± 3.27^b^	35.13 ± 3.82^b^	36.04 ± 0.27^b^
n-3PUFA	16.37 ± 0.29^a^	15.77 ± 0.87^a^	8.82 ± 1.06^b^	10.19 ± 1.43^b^	10.47 ± 0.85^b^
n-6PUFA	23.39 ± 0.40^a^	24.23 ± 0.49^a^	25.55 ± 2.29^a^	22.90 ± 2.27^a^	22.57 ± 1.05^a^
n3/n6	0.70 ± 0.02^a^	0.65 ± 0.05^a^	0.35 ± 0.02^c^	0.44 ± 0.03^bc^	0.47 ± 0.06^b^
DHA/EPA	0.36 ± 0.03^a^	0.35 ± 0.01^a^	0.29 ± 0.04^ab^	0.27 ± 0.03^b^	0.25 ± 0.02^b^

Note: the data are represented as the mean ± standard deviation (*n* = 3). Values in the same row with different letters are significantly different (*P* < 0.05). SFA: total saturated fatty acids; MUFA: total monounsaturated fatty acids; PUFA: total polyunsaturated fatty acids; n-3PUFA: n-3 polyunsaturated fatty acids; n-6PUFA: n-6 polyunsaturated fatty acids; 12:0: lauric acid; 14:0: myristic acid; 15:0: pentadecanoic acid; 16:0: palmitic acid; 17:0: heptadecanoic acid; 18:0: stearic acid; 20:0: peanut acid; 16:1: palmitoleic acid; 17:1: Cis-10-heptadecanoic acid; 18:1n9t: elaidic acid; 18:1n9c: oleic acid; 20:1n9: Cis-11-eicosenoic acid; 18:2n6c: linoleic acid; 18:3n6: *γ*-linoleic acid; 18:3n3: *α*-linoleic acid; 20:2: Cis-11, 14-eicosadienoic acid; 20:3n6: Cis-8, 11, 14-eicosatrienoic acid; 20:3n3: Cis-11, 14, 17-eicosatrienoic acid; 20:4n6: arachidonic acid; C20:5n3 (EPA): eicosapentaenoic acid; C22:2: Cis-13, 16 docosadienoic acid; 22:5n3 (DPA): docosapentaenoic acid; 22:6n3 (DHA): Docosahexaenoic acid.

**Table 8:** Fatty acid composition of female *E. sinensis* hepatopancreas in rice-crab coculture system at different developmental periods (% dry weight).

Fatty acid composition	Group				
	D0	D15	D31	D46	D62
12:0	0.12 ± 0.01^a^	0.13 ± 0.03^a^	0.07 ± 0.01^b^	0.09 ± 0.01^ab^	0.13 ± 0.01^a^
13:0	0.08 ± 0.01^ab^	0.12 ± 0.04^a^	0.05 ± 0.01^b^	0.07 ± 0.01^b^	0.07 ± 0.01^b^
14:0	1.88 ± 0.14^b^	2.29 ± 0.24^a^	1.28 ± 0.03^c^	1.24 ± 0.06^c^	1.29 ± 0.01^c^
15:0	0.82 ± 0.02^a^	0.79 ± 0.14^a^	0.45 ± 0.06^b^	0.58 ± 0.02^b^	0.56 ± 0.01^b^
16:0	34.11 ± 0.65^a^	29.19 ± 1.35^b^	20.91 ± 1.08^c^	23.16 ± 1.16^c^	22.40 ± 0.53^c^
17:0	0.18 ± 0.03^ab^	0.21 ± 0.02^a^	0.15 ± 0.01^b^	0.21 ± 0.02^a^	0.17 ± 0.02^ab^
18:0	4.55 ± 0.41^a^	3.99 ± 0.35^a^	2.59 ± 0.18^b^	3.09 ± 0.22^b^	2.94 ± 0.07^b^
20:0	0.41 ± 0.03^b^	0.49 ± 0.02^a^	0.33 ± 0.02^c^	0.33 ± 0.03^c^	0.31 ± 0.02^c^
21:0	—	—	0.03 ± 0.01^b^	0.02 ± 0.01^b^	0.12 ± 0.02^a^
22:0	0.74 ± 0.05^a^	0.67 ± 0.03^a^	0.49 ± 0.03^b^	0.42 ± 0.02^b^	0.31 ± 0.04^c^
24:0	—	—	0.24 ± 0.01a	0.22 ± 0.01a	0.18 ± 0.02b
SFA	42.89 ± 0.88^a^	37.87 ± 1.88^b^	26.60 ± 0.96^c^	29.44 ± 0.90^c^	28.48 ± 0.67^c^
14:1	0.13 ± 0.01^ab^	0.15 ± 0.03^ab^	0.10 ± 0.03^b^	0.14 ± 0.02^ab^	0.18 ± 0.02^a^
15:1	0.06 ± 0.01^a^	0.04 ± 0.01^ab^	0.03 ± 0.01^b^	0.04 ± 0.01^ab^	0.06 ± 0.01^a^
16:1	9.13 ± 0.33^ab^	6.90 ± 0.93^b^	8.92 ± 1.65^ab^	9.18 ± 0.76^ab^	9.50 ± 0.30^a^
17:1	0.05 ± 0.01^a^	0.05 ± 0.02^a^	0.05 ± 0.01^a^	0.06 ± 0.01^a^	0.05 ± 0.01^a^
18:1n9t	0.49 ± 0.02^a^	0.48 ± 0.04^a^	0.31 ± 0.03^c^	0.39 ± 0.02^b^	0.36 ± 0.03^bc^
18:1n9c	32.27 ± 0.33^a^	32.96 ± 0.09^a^	24.38 ± 0.46^c^	26.38 ± 0.76^b^	32.76 ± 0.40^a^
20:1n9	0.21 ± 0.02^b^	0.19 ± 0.01^b^	0.14 ± 0.01^c^	0.18 ± 0.02^bc^	0.27 ± 0.03^a^
MUFA	42.40 ± 0.29^a^	40.78 ± 1.08^a^	33.92 ± 1.89^b^	36.37 ± 1.56^b^	43.20 ± 0.67^a^
18:2n6t	0.04 ± 0.01^b^	0.04 ± 0.01^b^	0.03 ± 0.01^b^	0.05 ± 0.01^b^	0.14 ± 0.02^a^
18:2n6c	10.12 ± 0.84^c^	14.68 ± 2.54^b^	23.56 ± 0.63^a^	21.36 ± 0.85^a^	21.35 ± 1.13^a^
18:3n6	0.87 ± 0.05^b^	1.01 ± 0.12^ab^	0.56 ± 0.12^c^	1.20 ± 0.12^a^	0.83 ± 0.06^b^
18:3n3	1.56 ± 0.23^c^	2.36 ± 0.20^c^	6.22 ± 0.67^a^	4.23 ± 0.64^b^	2.50 ± 0.08^c^
20:2	0.36 ± 0.06^d^	0.66 ± 0.09^c^	1.14 ± 0.05^ab^	1.34 ± 0.11^a^	1.03 ± 0.06^b^
20:3n6	0.06 ± 0.02^a^	0.07 ± 0.01^a^	0.05 ± 0.01^a^	0.06 ± 0.01^a^	0.06 ± 0.01^a^
20:3n3	0.38 ± 0.07^ab^	0.43 ± 0.05^ab^	0.48 ± 0.02^a^	0.45 ± 0.06^ab^	0.33 ± 0.04^b^
20:4n6	0.37 ± 0.07^d^	1.23 ± 0.13^c^	4.71 ± 0.15^a^	3.16 ± 0.47^b^	0.69 ± 0.03^cd^
20:5n3 (EPA)	0.51 ± 0.09^d^	0.53 ± 0.07^d^	1.94 ± 0.04^a^	1.50 ± 0.19^b^	0.88 ± 0.07^c^
22:2	0.42 ± 0.07^a^	0.34 ± 0.03^ab^	0.45 ± 0.07^a^	0.28 ± 0.03^b^	0.28 ± 0.04^b^
22:5n3 (DPA)	—	—	0.13 ± 0.02^b^	0.23 ± 0.03^a^	0.06 ± 0.01^c^
22:6n3 (DHA)	—	—	0.20 ± 0.02^b^	0.34 ± 0.04^a^	0.18 ± 0.02^b^
PUFA	14.71 ± 0.81^e^	21.35 ± 2.96^d^	39.48 ± 0.97^a^	34.20 ± 0.85^b^	28.33 ± 1.27^c^
n-3PUFA	2.46 ± 0.35^d^	3.33 ± 0.31^cd^	8.97 ± 0.66^a^	6.75 ± 0.91^b^	3.95 ± 0.17^c^
n-6PUFA	11.47 ± 0.88^d^	17.03 ± 2.54^c^	28.91 ± 0.62^a^	25.82 ± 0.55^ab^	23.07 ± 1.08^b^
n3/n6	0.21 ± 0.04^bc^	0.20 ± 0.01^bc^	0.31 ± 0.03^a^	0.26 ± 0.04^b^	0.17 ± 0.00^c^
DHA/EPA	—	—	0.10 ± 0.01^b^	0.23 ± 0.03^a^	0.21 ± 0.02^a^

Note: the data are represented as the mean ± standard deviation (*n* = 3). Values in the same row with different letters are significantly different (*P* < 0.05). “-” represents no data for this period. SFA: total saturated fatty acids; MUFA: total monounsaturated fatty acids; PUFA: total polyunsaturated fatty acids; n-3PUFA: n-3 polyunsaturated fatty acids; n-6PUFA: n-6 polyunsaturated fatty acids; 12:0: lauric acid; 13:0: tridecanoic acid; 14:0: myristic acid; 15:0: pentadecanoic acid; 16:0: palmitic acid; 17:0: heptadecanoic acid; 18:0: stearic acid; 20:0: peanut acid; 21:0: hexadecanoic acid; 22:0: behenic acid; 24:0: tetracosanoic acid; 14:1: myristoleic acid; 15:1: Cis-10-pentadenoic acid; 16:1: palmitoleic acid; 17:1: Cis-10-heptadecanoic acid; 18:1n9t: elaidic acid; 18:1n9c: oleic acid; 20:1n9: Cis-11-eicosenoic acid; 18:2n6t: linolelaidic; 18:2n6c: linoleic acid; 18:3n6: *γ*-linoleic acid; 18:3n3: *α*-linoleic acid; 20:2: Cis-11, 14-eicosadienoic acid; 20:3n6: Cis-8, 11, 14-eicosatrienoic acid; 20:3n3: Cis-11, 14, 17-eicosatrienoic acid; 20:4n6: arachidonic acid; C20:5n3 (EPA): eicosapentaenoic acid; C22:2: Cis-13, 16 docosadienoic acid; 22:5n3 (DPA): docosapentaenoic acid; 22:6n3 (DHA): docosahexaenoic acid.

**Table 9:** Fatty acid composition of female *E. sinensis* ovary in rice-crab coculture system at different developmental periods (% dry weight).

Fatty acid composition	Group		
	D31	D46	D62
12:0	0.04 ± 0.01^a^	0.05 ± 0.01^a^	0.04 ± 0.01^a^
14:0	0.52 ± 0.03^a^	0.50 ± 0.04^a^	0.44 ± 0.11^a^
15:0	0.31 ± 0.04^a^	0.30 ± 0.02^a^	0.36 ± 0.06^a^
16:0	16.72 ± 0.38^c^	22.09 ± 0.42^a^	20.45 ± 0.31^b^
17:0	0.15 ± 0.02^b^	0.91 ± 0.06^a^	0.85 ± 0.06^a^
18:0	2.68 ± 0.25^c^	7.53 ± 0.07^a^	6.58 ± 0.47^b^
20:0	0.11 ± 0.02^b^	0.15 ± 0.02^a^	0.14 ± 0.01^a^
21:0	0.26 ± 0.03^a^	0.07 ± 0.01^b^	0.27 ± 0.2^a^
22:0	0.05 ± 0.01^b^	0.09 ± 0.02^a^	0.10 ± 0.01^a^
SFA	20.84 ± 0.57^c^	31.70 ± 0.35^a^	29.23 ± 0.51^b^
16:1	11.28 ± 0.26^a^	4.43 ± 0.15^b^	3.70 ± 0.29^c^
17:1	0.42 ± 0.03^b^	0.49 ± 0.03^ab^	0.60 ± 0.07^a^
18:1n9t	0.31 ± 0.03^a^	0.17 ± 0.01^b^	0.18 ± 0.02^b^
18:1n9c	25.86 ± 0.30^a^	26.16 ± 0.60^a^	26.76 ± 0.29^a^
20:1n9	0.08 ± 0.01^b^	0.80 ± 0.10^a^	0.75 ± 0.04^a^
22:1n9	0.08 ± 0.01^a^	0.13 ± 0.02^a^	0.07 ± 0.01^a^
24:1n9	0.05 ± 0.01^b^	0.13 ± 0.02^a^	0.14 ± 0.02^a^
MUFA	38.06 ± 0.36^a^	30.25 ± 0.55^c^	32.20 ± 0.19^b^
18:2n6t	0.08 ± 0.01^b^	0.20 ± 0.02^a^	0.19 ± 0.01^a^
18:2n6c	22.26 ± 0.89^a^	16.11 ± 0.61^c^	20.26 ± 0.76^b^
18:3n6	0.33 ± 0.02^a^	0.19 ± 0.02^b^	0.29 ± 0.04^a^
18:3n3	5.75 ± 0.16^a^	0.06 ± 0.01^b^	0.05 ± 0.01^b^
20:2	1.15 ± 0.06^b^	2.34 ± 0.21^a^	1.49 ± 0.27^b^
20:3n6	0.13 ± 0.03^b^	0.43 ± 0.05^a^	0.37 ± 0.04^a^
20:3n3	0.93 ± 0.07^a^	0.55 ± 0.05^c^	0.74 ± 0.07^b^
20:4n6	4.58 ± 0.15^b^	6.32 ± 0.39^a^	5.90 ± 0.21^a^
20:5n3 (EPA)	3.03 ± 0.15^c^	6.58 ± 0.16^a^	5.64 ± 0.59^b^
22:2	0.27 ± 0.03^a^	0.23 ± 0.03^a^	0.22 ± 0.04^a^
22:5n3 (DPA)	0.92 ± 0.07^a^	0.82 ± 0.04^a^	0.86 ± 0.05^a^
22:6n3 (DHA)	1.69 ± 0.09^c^	2.21 ± 0.03^b^	2.57 ± 0.18^a^
PUFA	41.11 ± 0.57^a^	36.04 ± 0.38^c^	38.58 ± 0.69^b^
n-3PUFA	12.31 ± 0.18^a^	10.22 ± 0.13^b^	9.89 ± 0.42^b^
n-6PUFA	27.38 ± 0.70^a^	25.24 ± 0.28^b^	27.00 ± 0.70^a^
n3/n6	0.45 ± 0.02^a^	0.40 ± 0.01^b^	0.36 ± 0.03^b^
DHA/EPA	0.56 ± 0.01^a^	0.34 ± 0.01^b^	0.46 ± 0.08^a^

Note: the data are represented as the mean ± standard deviation (*n* = 3). Values in the same row with different letters are significantly different (*P* < 0.05). SFA: total saturated fatty acids; MUFA: total monounsaturated fatty acids; PUFA: total polyunsaturated fatty acids; n-3PUFA: n-3 polyunsaturated fatty acids; n-6PUFA: n-6 polyunsaturated fatty acids; 12:0: lauric acid; 14:0: myristic acid; 15:0: pentadecanoic acid; 16:0: palmitic acid; 17:0: heptadecanoic acid; 18:0: stearic acid; 20:0: peanut acid; 21:0: hexadecanoic acid; 22:0: behenic acid; 16:1: palmitoleic acid; 17:1: Cis-10-heptadecanoic acid; 18:1n9t: elaidic acid; 18:1n9c: oleic acid; 20:1n9: Cis-11-eicosenoic acid; 22:1n9: erucic acid; 24:1n9: nervous acid; 18:2n6t: linolelaidic; 18:2n6c: linoleic acid; 18:3n6: *γ*-linoleic acid; 18:3n3: *α*-linoleic acid; 20:2: Cis-11, 14-eicosadienoic acid; 20:3n6: Cis-8, 11, 14-eicosatrienoic acid; 20:3n3: Cis-11, 14, 17-eicosatrienoic acid; 20:4n6: arachidonic acid; C20:5n3 (EPA): eicosapentaenoic acid; C22:2: Cis-13, 16 docosadienoic acid; 22:5n3 (DPA): docosapentaenoic acid; 22:6n3 (DHA): docosahexaenoic acid.

## Data Availability

The data supporting this study's findings are available from the corresponding authors upon reasonable request.
